# Avian Intestinal Mucus Modulates *Campylobacter jejuni* Gene Expression in a Host-Specific Manner

**DOI:** 10.3389/fmicb.2018.03215

**Published:** 2019-01-07

**Authors:** Torey Looft, Guohong Cai, Biswa Choudhury, Lisa X. Lai, John D. Lippolis, Timothy A. Reinhardt, Matthew J. Sylte, Thomas A. Casey

**Affiliations:** ^1^Food Safety and Enteric Pathogens Research Unit, United States Department of Agriculture, National Animal Disease Center, Agricultural Research Service, Ames, IA, United States; ^2^Crop Production and Pest Control Research Unit, United States Department of Agriculture, Agricultural Research Service, West Lafayette, IN, United States; ^3^GlycoAnalytics Core, University of California, San Diego, San Diego, CA, United States; ^4^Ruminant Diseases and Immunology Research Unit, Agricultural Research Service, United States Department of Agriculture, National Animal Disease Center, Ames, IA, United States

**Keywords:** *Campylobacter jejuni*, mucus, gene expression, RNAseq, antisense RNA

## Abstract

*Campylobacter jejuni* is a leading cause of bacterial foodborne illness in humans worldwide. However, *C. jejuni* naturally colonizes poultry without causing pathology where it resides deep within mucus of the cecal crypts. Mucus may modulate the pathogenicity of *C. jejuni* in a species-specific manner, where it is pathogenic in humans and asymptomatic in poultry. Little is known about how intestinal mucus from different host species affects *C. jejuni* gene expression. In this study we characterized the growth and transcriptome of *C. jejuni* NCTC11168 cultured in defined media supplemented with or without mucus isolated from avian (chicken or turkey) or mammalian (cow, pig, or sheep) sources. *C. jejuni* showed substantially improved growth over defined media, with mucus from all species, showing that intestinal mucus was an energy source for *C. jejuni*. Seventy-three genes were differentially expressed when *C. jejuni* was cultured in avian vs. mammalian mucus. Genes associated with iron acquisition and resistance to oxidative stress were significantly increased in avian mucus. Many of the differentially expressed genes were flanked by differentially expressed antisense RNA asRNA, suggesting a role in gene regulation. This study highlights the interactions between *C. jejuni* and host mucus and the impact on gene expression, growth and invasion of host cells, suggesting important responses to environmental cues that facilitate intestinal colonization.

**IMPORTANCE**

*Campylobacter jejuni* infection of humans is an important health problem world-wide and is the leading bacterial cause of foodborne illnesses in U.S. The main route for exposure for humans is consumption of poultry meat contaminated during processing. *C. jejuni* is frequently found in poultry, residing within the mucus of the intestinal tract without causing disease. It is not clear why *C. jejuni* causes disease in some animals and humans, while leaving birds without symptoms. To understand its activity in birds, we characterized *C. jejuni* responses to poultry mucus to identify genes turned on in the intestinal tract of birds. We identified genes important for colonization and persistence within the poultry gut, turned on when *C. jejuni* was exposed to poultry mucus. Our findings are an important step in understanding how *C. jejuni* responds and interacts in the poultry gut, and may identify ways to reduce *C. jejuni* in birds.

## Introduction

*Campylobacter jejuni* is an important human pathogen, causing up to 400 million infections a year world-wide (Ruiz-Palacios, 2007). According to the CDC, *Campylobacter* ranked the highest among the causes of foodborne illnesses in U.S. in 2016 (Marder, 2017). Enteritis from *Campylobacter* results in moderate to severe fever, abdominal pain, and diarrhea. Infections can also lead to serious complications, such as Guillain Barré syndrome (Epps et al., 2013). *C. jejuni* is considered an avian commensal and poultry are the main reservoir for human exposure, however some infections originate from livestock (Epps et al., 2013). Understanding how *C. jejuni* interacts within the host-intestinal environment will provide insights into how *C. jejuni* is so apt at colonizing the avian gut, and thus, may identify targets for limiting commensal carriage.

*C. jejuni* colonizes the avian intestinal tract, with densities reaching 10^10^ colony forming units (CFUs) per gram of intestinal contents (Young et al., 2007). Despite colonizing at such a high level, naturally colonized *C. jejuni* doesn't cause disease in poultry nor does it adhere or invade the intestinal epithelium (Larson et al., 2008). *C. jejuni* preferentially colonizes the mucus layer that lines the intestinal tract, where it resides, deep within the cecal crypts of birds (Epps et al., 2013). Its motility and helical shape aids *C. jejuni* movement through the thick mucus layer to reach these mucosal microenvironments favorable for growth (Lertsethtakarn et al., 2011; Stahl et al., 2016). Once there, *C. jejuni* has to survive harsh environmental conditions that include oxidative and nitrosative stress, iron limitation, and host defense peptides (Hofreuter, 2014). Mucus colonization is required by *C. jejuni* to cause disease. Previous studies suggest that host intestinal mucus, rather than host epithelial cells, impact *Campylobacter* phenotypes (Alemka et al., 2010; Ganan et al., 2013). In addition, *C. jejuni* is chemotactic for mucin and its components, such as L-fucose (Hugdahl et al., 1988), and chicken mucus impacts DNA supercoiling and motility (Shortt et al., 2016). When exposed to purified intestinal mucin from many different host sources, *C. jejuni* displays tropism for and preferential binding to avian mucins (Naughton et al., 2013). Furthermore, purified chicken mucus has been shown to reduce *C. jejuni* adherence and invasion to intestinal epithelial cells *in vitro*, compared to human mucus (Byrne et al., 2007). *Campylobacter*'s interactions with host mucins are highly specific, even showing differences among mucins isolated from different chicken intestinal compartments (Alemka et al., 2010; Naughton et al., 2013). These specific responses to avian mucus suggest adaptations to colonization and persistence within the avian gut.

In this study we characterized *C. jejuni* NCTC 11168 growth, invasion of intestinal epithelial cells, and transcriptome after exposure to purified mucus from avian and mammalian hosts. Avian and mammalian mucus sources differentially impacted gene expression that may reflect *Campylobacter*'s ability to colonize each animal intestinal tract, including the upregulation of iron acquisition and oxidative stress genes in avian mucus. Non-coding antisense RNAs were differentially detected between avian and mammalian hosts, and may be in response to environmental cues. These asRNA may be evidence of pervasive transcription in *C. jejuni* because they appeared to be generated by transcriptional read-through events that cross gene boundaries. These data suggest that *C. jejuni* alters its gene expression in the presence of avian mucus in ways that may benefit intestinal colonization. Understanding the molecular mechanisms of how *C. jejuni* colonizes mucus may suggest approaches to prevent colonization.

## Methods

### Mucus Source and Isolation

Intestinal mucus was isolated from the small intestine (duodenum, jejunum, and ileum) of healthy chickens, turkeys, pigs, sheep, or cows euthanized at the National Animal Disease Center, Ames Iowa, in accordance with a protocol approved by the Institutional Animal Care and Use Committee. Animal sources of mucus included: 10 turkeys (2 years old), 20 chickens (14 weeks old), 5 sheep (8 months old), five pigs (ranged from 2 to 4 years old), and one cow (around 2 years old). The mucosal surfaces were gently rinsed with sterile PBS to remove ingesta, and subsequently scraped with sterile microscope slides for collection of mucus. Mucus from respective animal species was pooled and lyophilized. Each mucus prep was purified to enrich the mucus glycans, as previously described (Martens et al., 2008). Briefly, lyopholized crude mucosal scrapings were suspended at 25 g/L in 100 mM Tris pH 7.4. Proteinase K was added (100 mg/L) to each sample and incubated 16–20 h at 65°C. Samples were then centrifuged at 21,000 × g for 30 min at 4°C and the supernatant was saved. NaOH was added to 0.1 M and NaBH4 to 1M and the solution was incubated 16–20 h at 55°C. HCl (12M) was added until pH reached 7.0. Mucus preps were centrifuged at 21,000 × g for 30 min at 4°C and supernatants were saved and filtered stepwise to clarify (5 μm → 3 μm → 1 μm → 0.65 μm → 0.45 μm → 0.22 μm). Mucus preps were dialyzed at 1 kDa cutoff against ddH2O to remove salts. Tris was added to the glycans at 50 mM (pH 7.4) and passed over DEAE-Cellulose (CAS 9013-34-7, Santa Cruz Biotechnology) columns equilibrated in 50 mM Tris pH 7.4 and the flow-through was collected. The resulting fractions were dialyzed at 1 kDa cutoff against ddH2O and lyophilized for downstream use.

### Mucus Composition

Glycan analysis of each mucus preparation was performed at the University of California, San Diego Glycotechnology Core facility. Analysis of the monosaccharide and sialic acid components of the glycans was done using high performance anion exchange chromatography with pulsed amperometric detection (HPAEC-PAD) and Reverse phase HPLC Fluorescence, respectively. Profiling of isolated glycans was done by MALDI-TOF and analyzed using the GlycoWorkbench Software Tool (Damerell et al., 2012).

Bradford protein assays were performed on mucus from each host to quantitate the total protein in each sample (Bradford, 1976). A total of 10 μg of each mucus preparation was run on SDS gel and stained with Coomassie blue and then SYPRO Ruby. Mass spectroscopy was performed, as described previously (Reinhardt et al., 2012), on each host mucus preparation (10% wt/vol) to characterize the proteins present in the mucus glycan preparation.

Iron quantification was performed for each host mucus sample using the EnzyX Iron (II) colorimetric assay (Assay Biotechnology Sunnyvale, CA). Samples were run at 1, 2, and 10% wt/vol (w/v), following the manufacturer's recommendations.

### *C. jejuni* Growth on Mucus

*C. jejuni* NCTC 11168 was obtained from the American Type Culture Collection (ATCC 700819) and grown at 42°C under microaerophilic conditions (85% N_2_, 5% O_2_, and 10% CO_2_) in MCLMAN (medium cysteine leucine methionine aspartic acid niacinamide) broth medium (Alazzam et al., 2011) for maintenance. Growth curves were generated (O.D._600_) by culturing *C. jejuni* NCTC11168 using the Bioscreen growth curve system (Growth Curves USA, Piscataway, NJ), with measurements taken each hour for 60 h. Cultures (10 replicates of each medium) were grown in 300 μl MCLMAN medium supplemented with 0.5% (w/v) purified mucus from chickens, turkeys, pigs, sheep, or cows, as well as MCLMAN without mucus.

### Adherence and Invasion of Host Epithelial Cells

Human embryonic INT-407 intestinal epithelial cells were used to test adherence and invasion by *C. jejuni* (Negretti and Konkel, 2017), with the following modifications. In some experiments, INT-407 cells were infected with *C. jejuni* and turkey or pig mucus was added only during the time of infection, and in others, mucus was present while culturing *C. jejuni*, as well as during the infection. The day before the assay, 1.5 × 10^5^ INT-407 cells were cultured at 37°C in 5% CO_2_ environment in wells of a 24 well plate using DMEM with 10% FBS containing penicillin and streptomycin (1X) and 10 mM HEPES buffer. For each treatment, INT-407 cells were plated in quadruplicate. The next day, cells were washed 3X in HBSS buffer containing calcium and magnesium prior to adding the inoculum. *C. jejuni* was grown on Mueller-Hinton plates at 42°C in a microaerophilic gas environment. At least 5 colonies were inoculated into the broth phase of biphasic liquid and agar (2% w/v) MCLMAN media. In some cases, the liquid phase of the biphasic media contained 0.5% (w/v) turkey or pig mucus. Inoculum was statically cultured for 48 h at 42°C in a microaerophilic gas environment. Motility of the inoculum was assessed by dark field microscopy. The inocula were prepared by adjusting the OD_540_ to 0.03 (3 × 10^7^ cfu/mL) in HBSS containing 1% BSA (fraction V) with or without the addition of 0.5% (w/v) turkey or pig mucus. Adherence and invasion was also tested without the addition of mucus to the inocula. A MOI of approximately 100:1 was used by adding 500 μL of the inocula to each well. Infection was synchronized by centrifuging the plate at 800 × g for 5 min at room temperature. Cells were incubated for 3 h at 37°C with 5% CO_2_ cells were then washed 3X in 1mL of HBSS and lysed with the addition of 200 μL of Triton X-100 for 5 min at 37°C. Cell associated *C. jejuni* (adherent and invaded) were enumerated by serial dilution and culture on Mueller-Hinton plates. After 3 h incubation, invasion was measured by removing the media from cells plated and inoculated in the same manner as described above, washing cells 3X with HBSS, and adding 1 mL of DMEM containing 1% FBS and 250 μg/mL gentamicin back to each well. Cells were incubated for 3 h and then washed 3X in HBSS. Cells were lysed as described above, bacteria were enumerated, and reported as the number of invaded bacteria.

### mRNA Isolation and Sequencing

*C. jejuni* was grown, while shaking under microaerophillic conditions, in 5.0 ml of MCLMAN liquid medium supplemented with 0.5% (w/v) of each host mucus and MCLMAN without mucus (four *C. jejuni* culture replicates of each). After a period of 24 h, the cultures were removed from the incubator and RNAprotect (QIAGEN, Germantown, MD) was added to each culture followed by RNA extraction using the RNeasy extraction kit (QIAGEN), following the manufacturer's instructions. DNA was removed using Turbo DNase (Ambion, Austin, TX). Total RNA quality was assessed with the 2100 Bioanalyzer, using RNA Nano chips (Agilent Tech., Santa Clara, CA). Ribosomal RNA was removed using the RiboZero system according to the manufacturer's instructions (Illumina, San Diego, CA), to enrich for total mRNA. Libraries were constructed using directional TruSeq RNA library kit and were sequenced on a HiSeq 2500 sequencer using the 100-cycle, high output mode (Illumina Inc., San Diego, CA).

### Full Length mRNA Sequencing

Messenger RNA used for RNA-Seq analysis from *C. jejuni* grown on chicken mucus was sequenced using PacBio's long read technology to evaluate if antisense RNA resulted from pervasive transcription extending from adjacent upregulated mRNA. Total RNA was isolated as described above. Transcripts were polyadenylated followed by removal of rRNA using the RiboZero kit (Illumina, San Diego, CA, United States). After rRNA depletion, cDNA was synthesized and amplified using SMARTer cDNA system (Clontech Laboratories, Mountain View, CA, United States), and low and high molecular weight fractions were selected using the BluePippin instrument (Sage Science, Beverly, MA, United States). Low and high molecular weight libraries were sequenced using PACBIO RS II sequencer, implementing the Iso-Seq pipeline (Pacific Biosciences, Menlo Park, CA, United States).

### Sequence Analysis

Sequence data was imported into CLC Genomic workbench V 8.0, and mapped to the *C. jejuni* NCTC 11168 reference sequence NC_002163 (Gundogdu et al., 2007). Reads were iteratively mapped to the genome in the following order: Mapped to the gene regions in the sense orientation, then the unmapped reads were mapped in the gene regions in the antisense orientation, and finally any remaining reads were mapped to intergenic regions. Expression values were calculated using only reads that mapped uniquely to a single location on the reference genome. Empirical analysis of differential gene expression was performed using the *EdgeR* statistical test, implemented in CLC Genomic workbench (Robinson et al., 2010). The data were normalized by scaling to the mean for graphical representations and presentation. A custom Pearl script was written to identify and bin all examples of differentially expressed genes [fold change >4.0, False Discovery Rate corrected (*FDR) p* < 0.01] in the sense orientation that were adjacent in the genome to differentially expressed genes in the antisense orientation. The criteria for these antisense-sense associations required the differentially expressed gene to be either the same gene as the antisense gene, or adjacent to it in the genome. If an association was identified, the next gene in the genome was evaluated. This continued until our fold change and *FDR p-value* threshold was not met.

### Comparisons With Publicly Available Data

To improve our understanding of the relationship between asRNA, iron acquisition, and oxidative stress genes, data from a study evaluating *PerR* (global regulator for oxidative stress response) or *Fur* (global regulator for iron acquisition) single or double knockouts on *C. jejuni* 11168 gene expression under iron limited or replete conditions was analyzed (Butcher et al., 2015). Briefly, the cDNA libraries of the transcripts had been generated in a strand specific manner by the authors, but the antisense data was not evaluated for proximity or linkages to the sense transcripts. After downloading the reads from NCBI (accession number SRP044881), the directional reads were mapped and analyzed as described above. Antisense RNA and gene expression within our dataset were identified as *Fur* or *PerR* repressed based on comparisons with the results from the published gene knockout study.

### Data Presentation

Analysis was performed using CLC Genomic workbench V 8.0 and PRIMER7 software v7.0.13 (Gorley and Clarke, 2008). PAST statistical analysis software (Hammer et al., 2001) was used for Permutational multivariate analysis of variance (PERMANOVA) tests. Differences with a False Discovery Rate (FDR) corrected *P*-value < 0.01 with at least a 4-fold change in gene expression was considered to be significant. Data are deposited in NCBI's Short Read Archive (SRA) associated with BioProject PRJNA411826.

## Results

### Growth Curves

**Figure 1:**
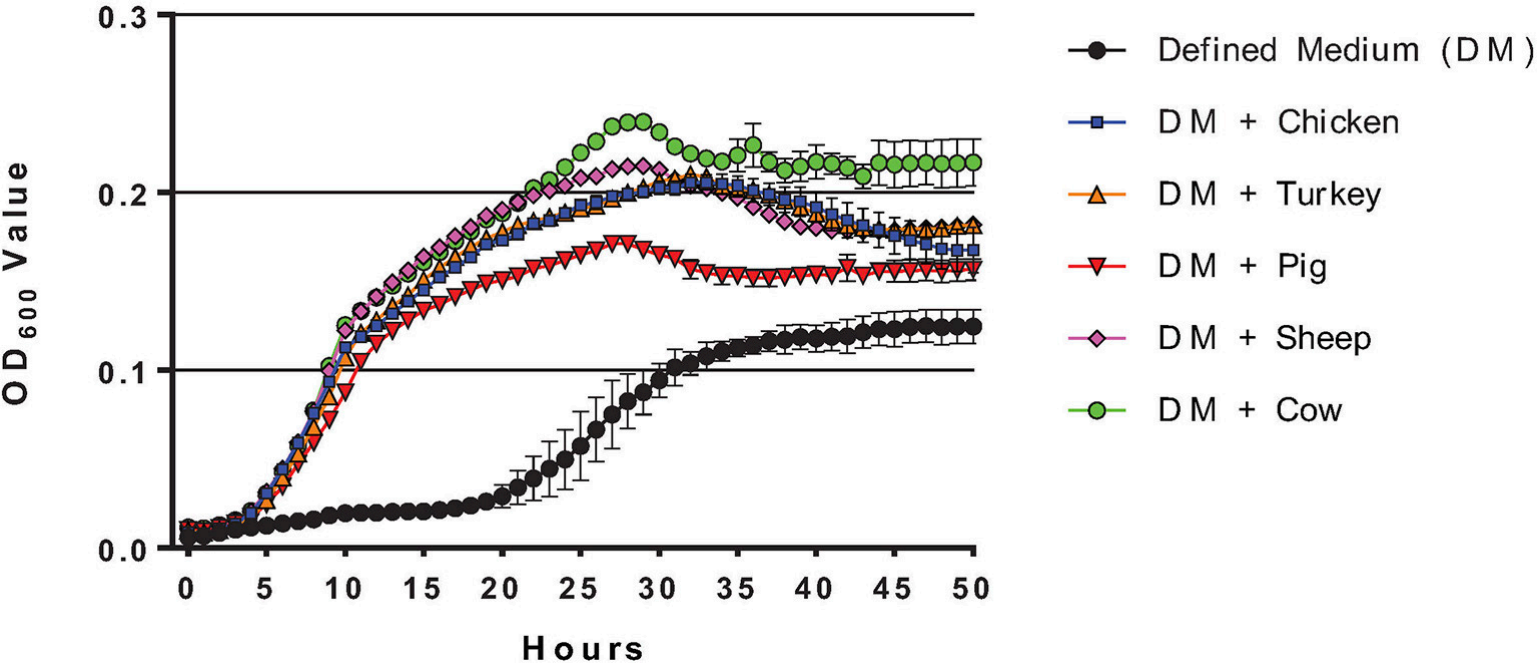
Growth curves of *C. jejuni* grown with and without mucus. Growth curves were generated from *C. jejuni* NCTC11168 grown in MCMAN defined media with, or without 0.5 % wt/vol mucus isolated from the small intestines of different animal species (Chicken, Turkey, Cow, Sheep, or Pig). Measurements were collected using a Bioscreen growth curve system (Growth Curves USA, Piscataway, NJ), with measurements taken each hour from 300 μl cultures. Standard errors are indicated for data points along the curve.

*C. jejuni* grown in MCLMAN broth supplemented with intestinal mucus from various species (0.5% w/v) showed a significant enhancement in growth and terminal O.D. (*P* < 0.001) compared to defined media alone, suggesting that mucus components were used as a growth substrate (Figure 1). The terminal O.D.s varied slightly among mucus sources, but no significant differences were detected between avian or mammalian mucus. Cultures supplemented with mucus reached terminal stationary phase after 28 h of growth, while it took 38 h on MCLMAN without mucus.

### Mucus Effect of Cell-Association and Invasion of Intestinal Epithelial Cells

**Figure 2:**
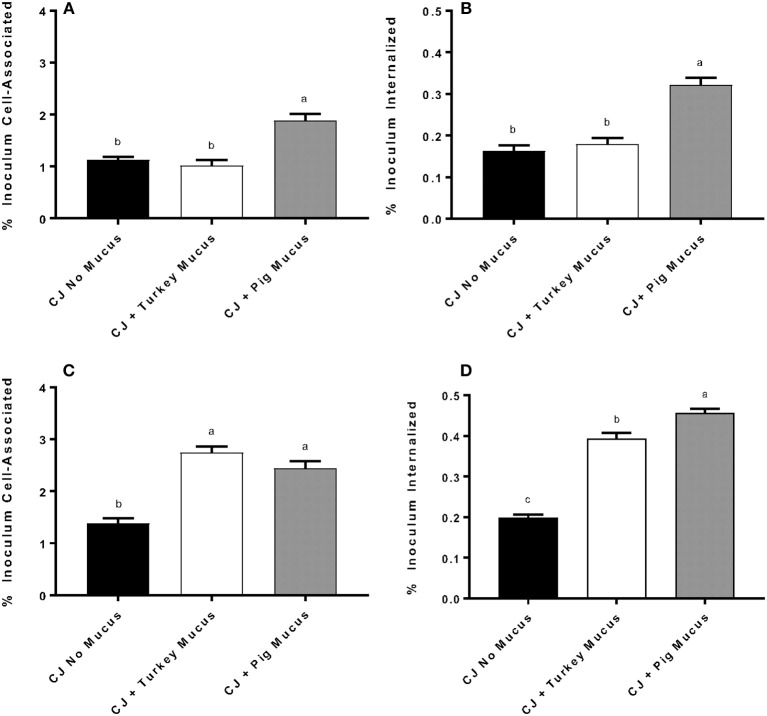
The animal source of mucus and its presence during growth or growth and adherence affect *C. jejuni* adherence and invasion of INT-407 cells. *C. jejuni* NCTC 11168 was cultured in defined medium with 0.5% (w/v) pig (CJ + pig mucus) or turkey (CJ + turkey mucus) mucus, or without mucus (CJ no mucus) and INT-407 cells were infected without mucus present during adherence **(A)** or invasion **(B)** phases. In other experiments, turkey or pig mucus was added to *C. jejuni* for growth in defined medium and during adherence **(C)** and invasion **(D)** phases. The MOI in each experiment was approximately 100:1. Data represent the mean ± SEM % cell-associated **(A,C)** or internalized **(B,D)** of four replicates and compared to the inoculum. Data were statistically analyzed using one-way ANOVA followed by a *post-hoc* multiple comparisons test (Tukey). Different letters in each panel represent significant differences (*P* < 0.05) in adherence or invasion of INT-407 cells by *C. jejuni* treated with or without mucus.

Addition of pig mucus [0.5% (w/v)] during the incubation stage of INT-407 cells with *C. jejuni* significantly enhanced (*P* < 0.001) the number of cell-associated and cell-invaded *C. jejuni*, compared to *C. jejuni* exposure with turkey mucus or without mucus (Figures 2A,B). In subsequent experiments, *C. jejuni* were cultured for 48 h in a defined media biphasic culture containing 0.5% (w/v) pig or turkey mucus, or no mucus in the liquid phase. Mucus was also present during the incubation stage of the assay. Culturing *C. jejuni* in either pig or turkey mucus significantly enhanced cell association and invasion, as compared to a control lacking mucus (Figures 2C,D). The number of invaded *C. jejuni* cultured in the presence of pig mucus was significantly different (*P* = 0.0013) compared to *C. jejuni* cultured in turkey mucus (Figure 2D).

### Mucus Composition

The crude mucus processing liberated mucus glycans from the protein backbone and enriched for O-linked neutral glycans (Martens et al., 2008). The total amount of protein present in each of the mucus preparations was low as determined by Bradford assays after purification [percentage/dry weight: chicken (0.12%), cow (0.15%), pig (0.08%), sheep (0.13%), and turkey (0.09%)], and compositional and structural analysis identified glycan sialic acid and differences between the avian and mammalian mucus (Table S1 and Figure S1). In each of the mucus samples, Fucose, N-Acetyl-galactosamine, N-Acetyl-glucosamine, Galactose, Glucose, and Mannose were present as glycan components. The sialic acids N-Glycolylneuraminic acid and N-Acetylneuraminic acid were both present in the glycans from cow, pig, and sheep mucus but N-Acetylneuraminic acid was the only sialic acid present in the chicken and turkey mucus (Table S1). Iron was not detectable in any of the mucus extractions by colorimetric assay (data not shown). The protein composition of each preparation was determined by MS/MS analysis (Table S2 and Figure S2), however no mucus proteins were identified, suggesting that glycans make up a large portion of the dry weight of the material isolated from each mucus (Figure S1).

### *C. jejuni* Transcriptome

In total, 417,448,571 sequence reads were obtained after quality filtration. 311,343,359 reads (74.6%) were mapped to protein coding regions in the sense orientation, 14,659,287 reads (3.5%) mapped to intergenic regions and 843,531 reads (0.2%) mapped to protein coding regions in the antisense orientation (Table 1).

**Table 1:** Sequencing statistics from *C. jejuni* mRNA RNA-seq data.

**Growth media**	**Total reads**	**uniquely-mapped reads to *C. jejuni* ref. genome (percent of total)**	**uniquely-mapped antisense reads to *C. jejuni* ref. genome (percent of total)**	**uniquely-mapped intergenic reads to *C. jejuni* ref. genome (percent of total)**
**SUMMARY OF *C. jejuni* RNA-SEQ DATA AND MAPPING STATISTICS**				
Defined media mean	17,354,770	8,166,591 (47%)	62,989 (0.36%)	557,194 (3.2%)
Chicken mucus mean	23,764,292	18,933,582 (80%)	46,384 (0.2%)	834,402 (3.5%)
Turkey mucus mean	15,026,164	12,162,509 (81%)	19,042 (0.13%)	578,640 (3.9%)
Cow mucus mean	20,173,514	16,927,895 (84%)	39,313 (0.19%)	678,646 (3.4%)
Pig mucus mean	15,187,045	10,964,828 (72%)	23,354 (0.15%)	475,245 (3.1%)
Sheep mucus mean	12,856,358	10,680,436 (83%)	19,802 (0.15%)	540,695 (4.2%)
Total (all replicates)	417,448,571	311,343,359	843,531	14,659,287

*The table show the number of reads generated on average for each sample by RNA-seq as well as their distribution among the mapped data*.

### Defined Media vs. All Mucus

**Figure 3:**
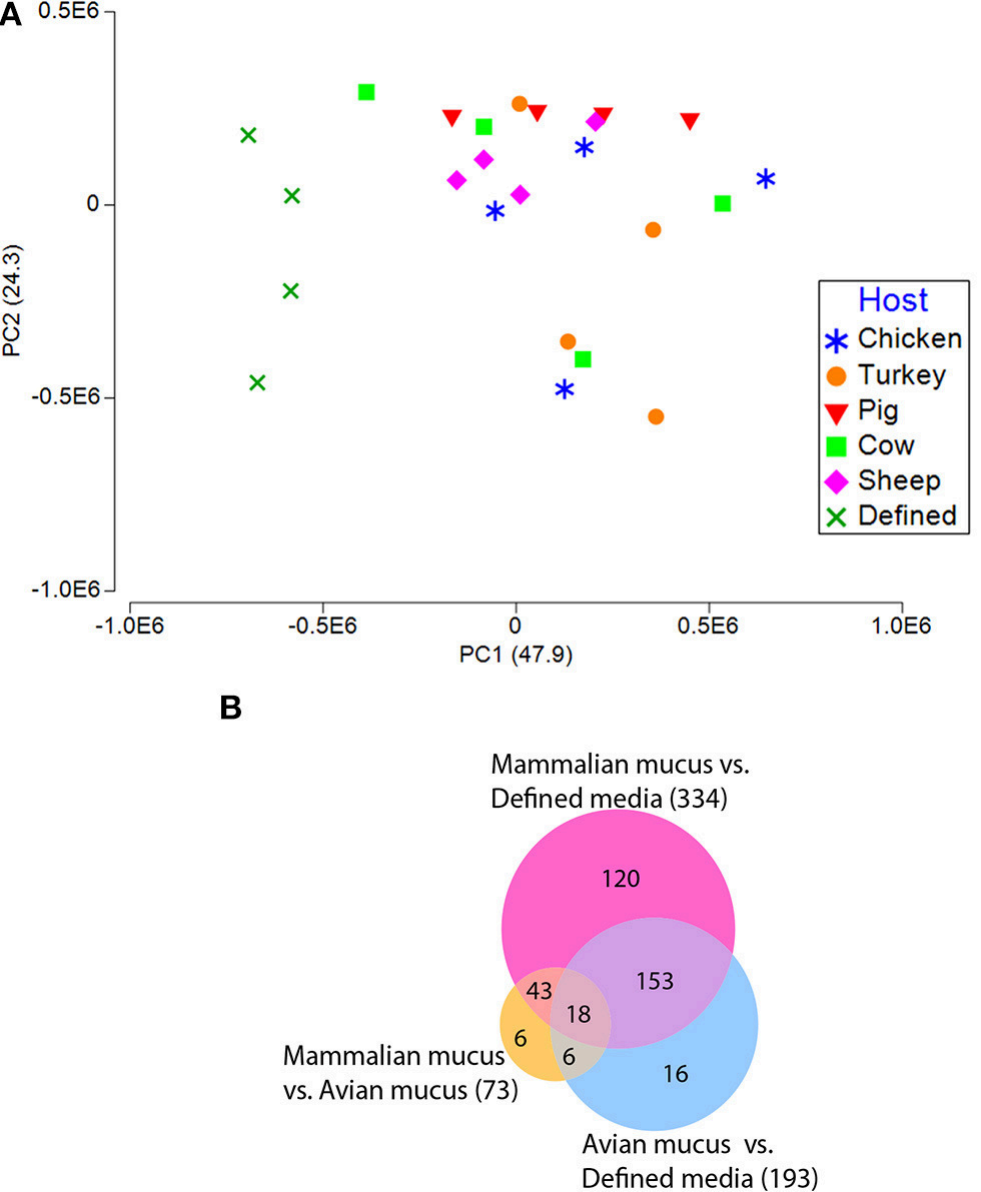
Transcriptomic profiles (sense) from *C. jejuni* grown on defined media with and without mucus isolated from avian and mammalian sources. **(A)** PCA plot generated from gene expression data of reads mapping to *C. jejuni* NCTC11168 coding regions of the reference genome in the sense orientation. **(B)** Venn diagram shows the number of differentially expressed genes (*fdr p* < 0.01, with > 4-fold difference) for all comparisons of avian mucus, mammalian mucus, and defined media.

Gene expression profiles from *C. jejuni* grown on defined media or defined media supplemented with intestinal mucus from different hosts significantly differed [Table S3, *P* < 0.001, Permutational multivariate analysis of variance test (PERMANOVA)]. A principal component analysis plot (PCA) of all transcriptomes showed differences in total transcriptomes between *C. jejuni* grown in the presence of mucus compared to without mucus (Figure 3A). The increased growth rate of all the mucus-containing cultures likely contributed to many of the expression differences observed. The mucus-containing cultures were in the late-log growth phase at the 24-h sampling, while the defined media were still in early-log phase. Because of these growth-rate differences, discussion of genes differentially expressed between mucus-containing and defined media will be limited to genes known to be impacted by the presence of mucus. A set of genes, part of the fucose utilization genetic island present in several *C. jejuni* strains including NCTC11168, were up regulated (9–19 fold) for all the mucus cultures. This gene cluster (*Cj0481*-*Cj0489*) includes fucose utilization proteins and a transporter.

### Differences Among Avian and Mammalian Host Mucus

Mucus-containing *C. jejuni* cultures were each in the late-log growth phase at the 24-h sampling for transcriptomic analysis (Figure 1). PCA plots of the *C. jejuni* transcriptomes didn't show clear separation among mucus from different hosts (Figure 3A). Despite overlap in expression profiles, there were 73 genes differentially expressed between *C. jejuni* grown in avian (chicken and turkey) and mammalian (pig, cow, and sheep) mucus (Table 2, Figure 3B). Most of the genes differentially expressed had increased expression in the avian mucus (71 genes). The 2 genes that were upregulated in the mammalian mucus were *rrc* and *fdxa* (both associated with oxidative stress response) and both were ~14-fold higher when compared to the avian mucus. Most of the *C. jejuni* genes upregulated in the avian mucus are involved in iron acquisition, oxidative response, or were membrane proteins. The genes that were most increased in the avian mucus, including *Cj1383c, chuC, Cj0177, exbD1, kata*, are involved in iron acquisition and oxidative stress response (all were over 100-fold higher in the avian mucus compared to mammalian mucus). In all, there were 35 genes associated with iron acquisition, 10 oxidative stress genes, 13 putative membrane proteins, and 13 genes of various functions (such as the energy taxis response gene *cetB*), upregulated in *C. jejuni* due to avian mucus, compared to mammalian mucus (Table 2).

**Table 2:** Genes differentially expressed between avian mucus (chicken and turkey) and mammalian mucus (cow, pig, and sheep).

	**Gene**	**Fold change**	**Description**
**GENES INCREASED IN *C. jejuni* GROWN IN AVIAN MUCUS RELATIVE TO MAMMALIAN MUCUS**			
**Iron uptake systems**	ceuB	−15	Iron compound ABC uptake transporter permease protein
	ceuC	−19	enterochelin uptake permease
	ceuD	−8	enterochelin uptake ATP-binding protein
	ceuE	−5	Enterochelin uptake periplasmic binding protein
	cfbpA	−19	Ferric iron ABC transporter, iron-binding protein
	cfbpB	−11	Ferric iron ABC transporter, permease protein
	cfbpC	−8	Putative iron-uptake ABC transport system ATP-binding
	cfrA	−89	Ferric receptor CfrA
	chuA	−87	Haemin uptake system outer membrane receptor
	chuB	−84	Haemin uptake system permease protein
	chuC	−159	Haemin uptake system ATP-binding protein
	chuD	−93	Haemin uptake, periplasmic haemin-binding protein
	Cj0177	−136	iron transport protein
	Cj0178	−74	Putative outer membrane siderophore receptor
	Cj0241c	−9	Hemerythrin domain protein
	Cj0818	−51	lipoprotein
	Cj0819	−68	Small hydrophobic protein
	Cj1383c	−179	hypothetical protein, co expressed with haemin uptake
	Cj1384c	−92	hypothetical protein, co expressed with haemin uptake
	Cj1397	−20	Ferrous iron transport protein A, putative
	Cj1587c	−7	ABC-type siderophore export system
	Cj1613c	−17	Putative heme oxygenase
	Cj1658	−14	High-affinity Fe2+/Pb2+ permease precursor
	Cj1660	−14	Fe2+ ABC transporter, substrate binding protein
	Cj1661	−15	Fe2+ ABC transporter, permease protein 1
	Cj1662	−14	Fe2+ ABC transporter, permease protein 2
	Cj1663	−11	Fe2+ ABC transporter, ATP-binding subunit
	exbB1	−89	Ferric siderophore transport system
	exbB2	−24	Ferric siderophore transport system
	exbD1	−134	Ferric siderophore transport system
	exbD2	−80	Biopolymer transport protein ExbD/TolR
	p19	−17	Periplasmic protein, high-affinity Fe2+ transport
	tonB1	−19	Ferric siderophore transport system, periplasmic binding
	tonB2	−25	Ferric siderophore transport system, periplasmic binding
	tonB3	−11	Ferric siderophore transport system, periplasmic binding
**Oxidative stress response**	perR	−11	Peroxide stress regulator
	trxB	−7	Thioredoxin reductase
	katA	−117	Catalase
	Cj1386	−44	Ankyrin-repeat containing protein
	Cj1308	−5	Putative acyl carrier protein
	Cj0556	−5	2-pyrone-4,6-dicarboxylic acid hydrolase
	Cj0379c	−8	Probable sulfite oxidase
	bioC	−4	O-methyltransferase
	ahpC	−11	Alkyl hydroperoxide reductase subunit C
	acpP2	−8	Putative acyl carrier protein
**Membrane associated**	Cj1668c	−5	Putative periplasmic protein
	Cj1665	−10	Possible lipoprotein thiredoxin
	Cj1664	−13	Possible periplasmic thiredoxin
	Cj1621	−5	Putative periplasmic protein
	Cj1406c	−6	Putative periplasmic protein
	Cj1381	−7	putative lipoprotein
	Cj1376	−15	periplasmic protein
	Cj1372	−5	Phospholipid ABC transporter protein
	Cj1207c	−4	Putative lipoprotein thiredoxin
	Cj1021c	−5	Putative periplasmic protein
	Cj0926	−6	Membrane protein
	Cj0378c	−9	Membrane protein
	Cj0176c	−15	Lipoprotein
**Other functions**	cetB	−5	Signal transduction protein, energy taxis
	Cj0120	−5	recombination protein RecO
	Cj0422c	−6	H-T-H containing protein
	Cj0444	−13	pseudo gene
	Cj0459c	−11	hypothetical protein
	Cj0717	−8	Glutathione-dependent thiol reductase
	Cj0878	−4	hypothetical protein
	Cj0880c	−5	hypothetical protein
	Cj0963	−5	DNA polymerase, bacteriophage-type
	Cj1208	−5	5-formyltetrahydrofolate cyclo-ligase
	dba	−14	disulfide bond formation protein
	dnaN	−8	DNA polymerase III beta subunit
	cgb	−5	Bacterial hemoglobin, nitrosative stress
**GENES INCREASED IN *C. jejuni* GROWN IN MAMMALIAN MUCUS RELATIVE TO AVIAN MUCUS**			
	fdxA	14	4Fe-4S ferredoxin, iron-sulfur binding
	rrc	14	Rubrerythrin, oxidative stress tolerance

*Differences are considered significant for genes that are differentially expressed greater than 4-fold with a FDR corrected P value less than 0.01. Genes with a negative fold change are increased in C. jejuni grown in the avian mucus, while positive fold change is increased in the mammalian mucus. Broad functional categories of differentially expressed genes are indicated to the right of genes*.

### Differences Among Each Host Mucus

All comparisons among *C. jejuni* grown on different host mucus identified genes differentially expressed between each animal mucus, with the exception of cow mucus compared to pig mucus, which had no significant differences (Table S4). Other than the defined media alone, chicken mucus was the strongest driver of *C. jejuni* gene expression differences, yielding 157, 152, 115, and 113 genes differentially expressed from cow, pig, sheep, and turkey mucus, respectively. The turkey mucus also diverged from the mammalian mucus resulting in 46, 49, and 47 differentially expressed genes from cow, pig, and sheep mucus, respectively. While there were no detected differences between cow and pig mucus, they had 23 and 21 genes differentially expressed with sheep mucus, respectively. Many of the genes identified with these comparisons reflect the avian or mammalian origin of the mucus. For example, iron acquisition and oxidative stress genes were again increased in *C. jejuni* grown in the chicken or turkey mucus, compared to pig, cow or sheep mucus. Some of these iron-associated genes were increased ~1,000 or ~2,000 fold (see chicken comparisons with cow or pig mucus, Table S4) in the avian mucus. The same 2 genes (*rrc* and *fdxa*) that were upregulated in the mammalian mucus when compared to avian, were also increased in the individual mammalian host comparisons with each avian host. Surprisingly, many of the same iron acquisition genes that were increased in the avian mucus, were also increased in the sheep mucus (i.e., *chuABD, tonB2, exbD2, exbB2, cfbpAB, p19, ceuB*, etc), when sheep was compared to the other mammalian mucus (pig and cow), highlighting differences among animal species.

### Antisense RNAs

**Figure 4:**
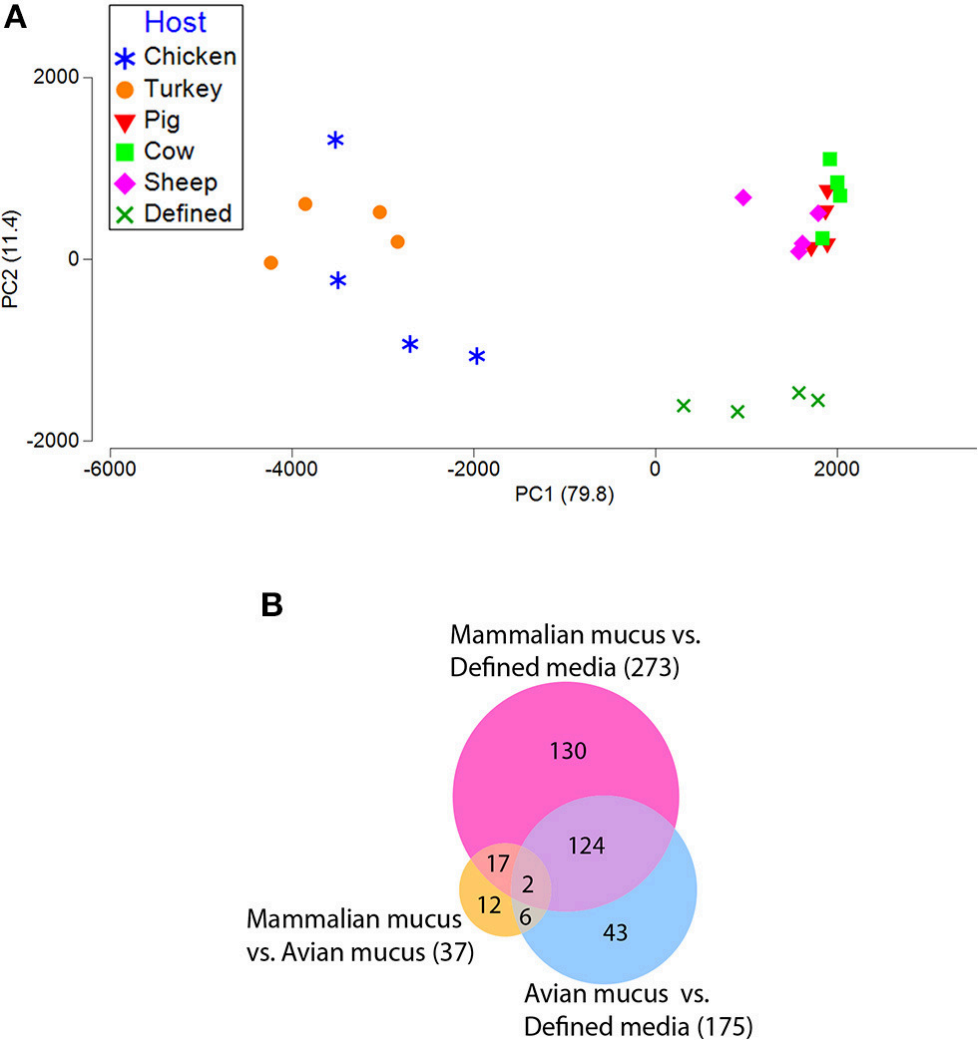
Antisense transcriptomic profiles among *C. jejuni* grown on avian mucus (chicken or turkey), mammalian mucus (cow, pig, or sheep), and the basal defined media without mucus. **(A)** PCA plot generated from antisense reads (map to non-coding strand of gene) from *C. jejuni* grown on defined media with and without mucus isolated from different host sources. **(B)** Venn diagram shows significant differences for all comparisons of avian mucus, mammalian mucus, and defined media for the antisense portion of the data (*fdr p* < 0.01, with > 4 fold difference).

Antisense sequences were separated from the *C. jejuni* transcriptome for parallel analysis. These transcripts were identified in the directional RNA-seq dataset by mapping the reads to genes in the antisense orientation. In all, 641 asRNAs, that mapped to the noncoding strand of genes, were identified in the dataset (had at least 10 reads). These asRNAs expression patterns segregated by source of mucus (host) in the *C. jejuni* growth media (Figure 4A). The *C. jejuni* grown on avian mucus (chicken or turkey) had asRNA expression profiles (PERMANOVA test) significantly different from the mammalian mucus' (cow, pig, or sheep); and both were different from that of the defined media, (*p* < 0.01), but there were no differences within groups (avian or mammalian). Many asRNAs were differentially present among *C. jejuni* grown in each host mucus as well as between defined media compared to all mucus types (Figure 4B). In many cases the asRNAs displayed atypical patterns, mapping to only portions of genes and intergenic regions.

Two *C. jejuni* genes (*prfA* and *flip*) had many antisense reads (relative to other asRNA) that mapped to the noncoding stand in the avian mucus cultures, and were also significantly increased in the avian mucus, compared to the mammalian mucus. Upon closer examination, it was observed that *prfA* and *flip*, while not differentially expressed in the sense orientation, were adjacent to differentially expressed genes in the sense orientation, within the same samples (*Cj1613c* next to *prfA* and *Cj0819* next to *fliP*). Global analysis of all of the sense and antisense data showed that many asRNAs that were upregulated in the avian transcriptomes, were adjacent coding mRNAs that were upregulated in those same samples (Table 3). This phenomenon was not only observed in the avian samples, but was often associated with upregulated genes.

**Table 3:** Association between differentially expressed antisense reads with adjacent differentially expressed genes between *C. jejuni* grown on avian or mammalian mucus.

**Antisense RNA (fold change)**	**Sense gene(s) (fold change)**	**Genome position relative to antisense RNA**
Cj0145 (−4.9)^**^	trxB (−7.2)^**^	1
trxA (−4.6)	trxB (−7.2)^**^	−1
cfbpA (−16)^*^	cfbpA (−19)^*^	0
	Cj0176c (−14.7)^*^	1
	Cj0177 (−137)^*^	2
	Cj0178 (−73.8)^*^	3
	exbB1 (−89.4)^*^	4
	exbD1 (−128.1)^*^	5
	tonB1 (−19.2)^*^	6
	cfbpB (−11.2)^*^	−1
	cfbpC (−7.6)^*^	−2
ahpC (−4)^**^	ahpC (−10.6)^**^	0
	fdxA (14)	−1
accA (−8.9)^*^	Cj0444 (−13.1)^*^	1
Cj0447 (−8.4)	Cj0444 (−13.1)	−1
Cj0752 (−6.9)^*^	tonB3 (−11.2)^*^	1
	cfrA (−87.4)^*^	2
tonB3 (−9.8)	tonB3 (−11.2)^*^	0
	cfrA (−87.4)^*^	1
cfrA (−5.8)^*^	cfrA (−87.4)^*^	0
	tonB3 (−11.2)^*^	−1
fliP (−40.6)^*^	Cj0819 (−59.5)^*^	−1
	Cj0818 (−50.5)^*^	−2
Cj0879c (−6)	Cj0880c (−4.6)	1
	Cj0878 (−4.8)	−1
Cj1309c (−7)	Cj1308 (−4.6)	−1
pldA (−10.7)	ceuB (−14.5)	1
	ceuC (−18.5)	2
	ceuD (−8)	3
	ceuE (−5.2)	4
tRNASer_1 (−4.6)	ceuE (−5.2)	−1
	ceuD (−8)	−2
	ceuC (−18.5)	−3
	ceuB (−14.5)	−4
Cj1381 (−12)^*^	Cj1381 (−7.1)	0
Cj1387c (−11.8)^**^	Cj1386 (−43.6)^**^	−1
	katA (−116)^**^	−2
	Cj1384c (−92)^*^	−3
	Cj1383c (−177.7)^*^	−4
cgb (−6.8)^*^	cgb (−4.7)	0
	Cj1587c (−6.5)^*^	1
prfA (−10.6)^*^	Cj1613c (−16.5)^*^	1
	chuA (−84.9)^*^	2
	chuB (−83.4)^*^	3
	chuC (−164.7)^*^	4
	chuD (−91.8)^*^	5
Cj1613c (−6.5)	Cj1613c (−16.5)^*^	0
	chuA (−84.9)^*^	1
	chuB (−83.4)^*^	2
	chuC (−164.7)^*^	3
	chuD (−91.8)^*^	4
Cj1618c (−270.3)^*^	chuD (−91.8)^*^	−1
	chuC (−164.7)^*^	−2
	chuB (−83.4)^*^	−3
	chuA (−84.9)^*^	−4
	Cj1613c (−16.5)^*^	−5
Cj1666c (−11.3)^*^	Cj1668c (−4.1)	1
	Cj1665 (−10.1)^*^	−1
	Cj1664 (−13)^*^	−2
	Cj1663 (−11.3)^*^	−3
	Cj1662 (−14.1)^*^	−4
	Cj1661 (−14.4)^*^	−5
	Cj1660 (−14.3)^*^	−6
	p19 (−16.4)^*^	−7
	Cj1658 (−13.4)^*^	−8

Table shows antisense RNAs that are differentially expressed (first column) between C. jejuni grown in avian or mammalian mucus, with fold change in parentheses (negative values indicate an increase in the avian mucus samples). The second column shows differentially expressed (sense) genes and fold change, found in the same sample the antisense RNA was identified. The criteria for these antisense-sense associations required the differentially expressed gene to be either the same gene as the antisense RNA, or adjacent to it in the genome (fdr p-value < 0.01, with >4 fold difference). If an association was identified, the next gene on genome was evaluated (relative positions to antisense in third column). This continued until our threshold was not met. Antisense RNA or sense genes with

(*) were confirmed to be Fur repressed and

(**) *) are confirmed to be PerR repressed after analysis of dataset from Butcher et al. (2015)*.

In addition to the positive associations among antisense and sense RNAs described above, negative associations among asRNA and their adjacent sense mRNA was observed with mucus comparisons with the defined media. The eight-gene fucose utilization operon (*Cj0481*-*Cj0489*), upregulated in all mucus cultures, had antisense reads mapping to the adjacent transcriptional regulator for the operon, *Cj0480c*, in the defined media cultures. Antisense RNA that mapped to *Cj0480c* was significantly increased while the rest of the operon was downregulated in the defined media. The close association between *C. jejuni* asRNAs and differentially expressed genes may suggest that these non-coding transcrips are associated to regulatory activities.

Many of the asRNAs upregulated in the avian mucus in this study were adjacent to genes associated with iron acquisition or oxidative stress response. To evaluate the role of the transcriptional regulators for iron acquisition (*Fur*) and oxidative stress response (*PerR*), public data were retrieved and re-analyzed from a report by Butcher et al. (2015) to evaluate the association between the asRNA and genes part of the *Fur* or *PerR* regulatory networks. In their study, Butcher et al. characterized *C. jejuni* NCTC11168 wild type, Δ*Fur*, Δ*PerR*, and Δ*Fur*Δ*PerR* mutants under both iron-replete and iron-limited conditions to identify the *Fur* and *PerR* regulons. In doing so, they identified the *Fur* or *PerR* regulated genes under iron limited and iron replete conditions. The researchers used strand-specific RNA-seq, allowing the sense or antisense nature of the RNAs to be preserved in the data. We identified many of the same asRNAs, observed in our dataset, in our re-analysis of their data, and confirmed the associations of the asRNA in our study are part of the *Fur* or *PerR* regulons for iron acquisition or oxidative stress protection, respectively. The differentially expressed genes and asRNAs shared with our study are noted with an asterisk on Table 3.

### Pervasive Transcription

We suspected that the detected asRNAs might be caused by delayed termination of mRNA, resulting in transcriptional read-through because the differentially expressed antisense reads correspond to genes arranged in the opposite direction. Read-through of RNA polymerase would create transcripts with an antisense portion if transcription continues into adjacent genes arranged in the opposite orientation. To test this, one of the replicates of *C. jejuni* grown in chicken mucus was subjected to full length cDNA sequencing on a PacBio instrument to evaluate the nature of the observed antisense RNAs. While the sequencing depth was low, we obtained sequences spanning regions where asRNA were adjacent to sense mRNAs. The most abundant asRNAs in the avian RNA-seq data mapped to the opposite stand of *prfA*, adjacent to the upregulated gene *Cj1613c* in the genome. Long reads spanning gene boundaries were observed in this region, suggesting that some antisense RNAs were being generated by failure of transcriptional termination to occur at gene boundaries, continuing into the non-coding stand of the open reading frame of the adjacent gene (Figure S3).

This transcriptional read-through did not always occur and did not appear to be proportional to transcriptional levels. For example, *porA* was the most highly expressed gene across all transcriptomes (1,151,741 reads average per sample, compared to 65,688 average reads mapping to *Cj1613c* in the avian samples). Immediately downstream of *porA* is *dnaJ*, in the opposite orientation as *porA* (similar to orientation of *Cj1613c* to *prfA*). Despite this, *dnaJ* only has on average only 5 antisense reads mapping to it in our dataset, compared to 6,264 antisense reads mapping to *prfA* in the avian mucus *C. jejuni* transcriptomes.

## Discussion

In this study, we described *C. jejuni* transcriptional responses to avian or mammalian intestinal mucus from different host species. Niche adaptation is expected for host-associate microbes and *C. jejuni's* preferred environment is the mucus along the mucosa of the avian gut (Epps et al., 2013). Several studies have identified phenotypic responses of *C. jejuni* to mucus' or mucins, but a full understanding of how *C. jejuni* responds to these environmental cues is still lacking (Ganan et al., 2013; Naughton et al., 2013; Shortt et al., 2016). Avian mucus was shown to modulate pathogenicity by reducing *C. jejuni* cellular adherence and invasion (Alemka et al., 2012), and our findings where mucus was added only during inoculation period concur with these prior findings. However, our adherence and invasion results differ from previous work when *C. jejuni* was cultured in the presence of either mammalian or avian mucus. This may be explained by the different intestinal cell lines used, as we used the INT-407 cell line, which is highly sensitive to *Campylobacter* adherence and invasion (Monteville et al., 2003). In addition, the previous study only addressed the effect of adding mucus to a rich tissue culture medium during the adherence and invasion assay and used up to 20% mucus (w/v) compared to 0.5% in the present study. Incubating INT-407 cells with *C. jejuni* with or without mucus in a nutrient deficient medium (HBSS with 1% BSA) was less likely to affect gene expression of *Campylobacter* when cultured in the defined medium with mucus. *C. jejuni* is chemotactic to and preferentially binds avian mucus over mucus from other hosts, but despite the prevalence in the avian gut, *C. jejuni* isn't associated with pathology in avian hosts (Naughton et al., 2014). In fact, *C. jejuni* recognizes and binds multiple glycan structures found within mucus, including terminal mannose, N-acetylneuraminic acid, galactose and fucose (chemotaxis and flagellar genes were upregulated in the mucus cultures in this study, but just below the 4-fold cutoff applied) (Day et al., 2009). Adaptations to the host environment are important for pathogen and commensal survival within the animal intestinal tract. We observed genes important for survival in this intestinal niche to be upregulated when *C. jejuni* is exposed to mucus.

Many genes were upregulated in the defined (minimal) media (208 genes), many of these differences may be related to the different growth phases of *C. jejuni* in defined media (early log) compared to the different mucus medias (each were late log), because *C. jejuni* is known to alter its expression profiles in each growth phase (Wright et al., 2009). Of the 33 genes increased in the mucus cultures, those increased the greatest were the genes part of the fucose utilization operon (*Cj0481*-*Cj0489*), a response shared regardless of mucus source. These genes have been shown to be necessary for growth on fucose which is an abundant sugar found in mucin (Muraoka and Zhang, 2011), and *C. jejuni* NCTC11168 is one of several *C. jejuni* strains known to have this operon and to utilize fucose (Muraoka and Zhang, 2011; Stahl et al., 2011). Strains of *C. jejuni* from human and avian sources show affinities for fucose, suggesting its importance for persistence in the gut (Day et al., 2013). Despite the broad importance of fucose for colonization of the gut, fucose permease (fucP) knockout mutants have a colonization defect in mammals but not in the avian gut, suggesting additional substrates support *C. jejuni* colonization of the avian gut (Stahl et al., 2011).

Many genes upregulated in *C. jejuni* grown in avian mucus, compared to mammalian mucus, have been shown to be essential for colonization of the avian gut. Specifically genes associated with resistance to oxidative stress (e.g., *perR, katA, ahpC, trxB*) and iron (heme) binding and transport (e.g., *ceuBCDE, cfbABC, chuABCD, exbB1, exbB2, exbD1, exbD2, p19, tonB1, tonB2, tonB3*) (Hofreuter, 2014; Butcher et al., 2015) were significantly increased in the avian mucus cultures. Interestingly, not all iron transport genes were increased in the avian mucus. *FeoB*, involved in ferrous iron acquisition wasn't differentially expressed (1.4-fold difference with the mammalian mucus) (Naikare et al., 2006). Studies with knockout mutants have established that loss of genes involved in protection against oxidative and nitrosative stress, and iron acquisition reduce or eliminate *C. jejuni* colonization of the chicken gut (Palyada et al., 2004, 2009). *C. jejuni* similarly responds to bile, perhaps as an environmental cue, upregulating oxidative stress response genes after exposure to bile salts (Negretti et al., 2017). Many of the genes upregulated in avian mucus were also identified in a previous transcriptomic study of *C. jejuni* in chickens,*in vivo* (Taveirne et al., 2013). These include the oxidative and nitrosative stress response genes *katA* and *cgb*, and iron acquisition genes *chuABCD* and *exbB-2*, and *exbD* (Taveirne et al., 2013). Iron acquisition genes CJJ81176_1649 (Cj1658) and p19 were similarly upregulated in *C. jejuni* strain 81-176 when exposed to chicken cecal extracts from chicken or fecal extracts from humans (Liu et al., 2018). Many of these same iron acquisition and oxidative stress pathways are also upregulated in *C. jejuni* during human infection (Crofts et al., 2018).

Dual oxidase (DUOX2) (El Hassani et al., 2005) and nicotinamide-adenine dinucleotide phosphate (NADPH) oxidase 1 (NOX1) (Szanto et al., 2005) are reactive oxygen species (ROS)-producing NADPH oxidases highly expressed in the lower intestinal epithelium, and serve as one of the innate effector mechanisms to prevent bacterial invasion of the mucosa. The ROS generated from DUOX2 and NOX1 alter capsule synthesis in *C. jejuni*, reducing its virulence (Corcionivoschi et al., 2012). *C. jejuni* uses heme-containing enzymes to counter the effects of host-derived hydrogen peroxide defenses (Alvarez et al., 2016). The interaction of mucus shed into the small intestinal lumen and *C. jejuni* may serve as an early signal to upregulate genes involved in resistance to oxidative stress, and adapt *C. jejuni* to survive within cecal mucus (Beery et al., 1988), in close proximity to ROS-generating epithelium.

Other mucosal colonizers have been characterized with similar responses to mucus, by upregulating essential colonization pathways. Transcriptomic comparisons from mucus-associated and intestinal lumen associated *Escherichia coli* from monocolonized mice showed very different functional pathways being upregulated in each environment (Li et al., 2015). While pathways such as glycerol metabolism and fatty acid degradation were upregulated in the lumen, genes belonging to the *Fur* regulon, including iron uptake and mobilization genes, and oxidative stress response genes were upregulated in the mucus. *C. jejuni* has also been shown to respond to microbial-derived short chain fatty acids within the intestinal lumen by modulating gene expression, leading to colonization (34).

*Pseudomonas aeruginosa*, an opportunistic pathogen and potential colonizer of mucus in the respiratory tract of cystic fibrosis patients, upregulates iron acquisition genes, peroxide-detoxifying enzymes and small regulatory RNAs when exposed to human respiratory mucus *in vitro* (Cattoir et al., 2013; Gi et al., 2015). Many of the genes upregulated in *C. jejuni* in the presence of avian mucus (*katA*, catalase; *ahpC*, alkyl hydroperoxide reductase subunit C; *trxB*, thioredoxin; *Cj1386c*, ankyrin containing protein) have homologus genes upregulated in *P aeruginosa* in human mucus (Cattoir et al., 2013). *Cj1386c* is downstream from *katA* and enhances catalase activity by trafficking heme to catalase (Flint et al., 2012). It is unclear if the upregulation of *katA* and *Cj1386* creates and iron demand, resulting in the upregulation of iron acquisition genes observed in this study. Oddly, superoxide dismutase (*sodB*) was not changed in *C. jejuni* in the avian mucus.

Here we show that antisense reads from *C. jejuni* grown in mucus from different host sources correlate with their respective host mucus environment [(avian; chicken or turkey), mammalian; (cow, pig, or sheep), or defined media; Figures 4A,B]. Several reports have described the existence of asRNAs in *C. jejuni* transcriptomes and have suggested roles in gene regulation because of the lack of many known transcriptional regulators in *C. jejuni* (Taveirne et al., 2013; Butcher et al., 2015). Antisense RNAs have also been observed upregulated in *C. jejuni in vivo*, suggesting important regulatory rolls in the intestinal environment compared to standard laboratory media (Taveirne et al., 2013). Of the 37 differentially expressed asRNA between avian and mammalian mucus cultures, 21 are adjacent to genes that were also differentially expressed. Most of these genes are regulated by one of two global regulators for iron acquisition or oxidative stress, *Fur* and *PerR*, respectively. Understanding this response is complicated by the overlapping regulons of *Fur* and *PerR*. We confirmed the association of these asRNA to the *Fur* or *PerR* regulons by re-analyzing the strand-specific data from a previously published study that evaluated *C. jejuni* NCTC11168 wild type, Δ*Fur*, and Δ*PerR* under both iron-replete and iron-limited conditions (Butcher et al., 2015). Both of these regulators repress expression of genes in their respective regulon. Data generated by Butcher et al. (Butcher et al., 2015) showed that Δ*Fur*, or Δ*PerR* mutants failed to repress asRNA production (upregulated in mutants), as well as the genes belonging to each regulon, when compared to wild type *C. jejuni* NCTC11168. Most of the asRNA and adjacent differentially expressed genes in our study appeared in their dataset (Table 3). How these asRNAs may associated with adjacent genes, and why they are impacted by avian mucus is not clear.

Information about longer transcripts or transcriptional read through is lost with the short reads obtained from RNA-seq. To examine long transcripts, we sequenced the mRNA from one of the samples using the PacBio platform to see if the observed asRNAs were small elements or part of a longer transcripts. We confirmed that some of these asRNAs are part of larger transcripts that span gene boundaries (low sequencing depth prevented us from evaluating all asRNA). With broad use of strand-specific RNA-seq, there have been many other reports of bacterial transcription crossing gene boundaries and starting or terminating mid-gene, including the non-coding strand resulting in antisense transcripts (Wade and Grainger, 2014; Bidnenko et al., 2017). This non-canonical transcription has been named “pervasive transcription” and has been observed in all domains of life (Berretta and Morillon, 2009; Lybecker et al., 2014). The role of pervasive transcription in bacteria is unknown and resulting transcripts remain uncharacterized, but it has been observed in many bacterial species (Lybecker et al., 2014; Wade and Grainger, 2014). Whether the identified asRNA results from pervasive transcription or have regulatory functions requires further experiments.

## Conclusion

*C. jejuni* has a commensalistic relationship with its avian hosts, yet can be pathogenic in the mammalian intestinal tract, including humans. In the avian gut, *C. jejuni* colonizes mucus in the intestinal crypts, without pathology or disease in the host. Previous studies suggest intestinal mucus may contribute to the different responses of *C. jejuni* in the diverse gut environments. We showed that avian mucus modulates *C. jejuni* adherence and invasion of host cells, and impacts transcriptomic response, different from that of *C. jejuni* grown in mammalian mucus. Many of the genes upregulated when *C. jejuni* was grown in avian mucus, are essential for colonization of the avian gut. Understanding how *C. jejuni* responds to and interacts with the avian gut environment may identify intervention targets for reducing *C. jejuni* prevalence in the food chain.

## Author Contributions

TL conceived and designed the study, as well as in drafted the manuscript. TL, BC, LL, JL, TR, and MS performed the experiments and collected data. TL, GC, and TC analyzed and interpreted the data. TC and MS revised the paper for important content.

### Conflict of Interest Statement

The authors declare that the research was conducted in the absence of any commercial or financial relationships that could be construed as a potential conflict of interest.
