# Supplementary material for: Avian Intestinal Mucus Modulates Campylobacter jejuni Gene Expression in a Host-Specific Manner
Source: Front Microbiol. 2019 Jan 7;9:3215. doi: 10.3389/fmicb.2018.03215 (PMC6338021; doi:10.3389/fmicb.2018.03215)
Supplement: Supplementary file 1 [file Table_1.pdf]

Supplementary table 1.Monosaccharide and sialic acid composition of purified mucus (ng/µg)

	Chicken Mucus	Turkey Mucus	Cow Mucus	Pig Mucus	Sheep Mucus
Fucose	15.29	21.63	14.09	13.69	10.31
N-Acetyl-galactosamine	5.3	7.22	3.11	13.64	6.08
N-Acetyl-glucosamine	42.72	54.25	33.18	28.96	20.5
Galactose	24.67	28.52	23.38	20.17	14.28
Glucose	5.15	9.43	4.06	18	5.3
Mannose	15.44	21.13	15.84	19.79	13.36
N-Glycolylneuraminic acid (sialic acid)	0	0	2	2.3	0.8
N-Acetylneuraminic acid (sialic acid)	2.7	6.1	0.6	2.2	1.5
