# Supplementary material for: Avian Intestinal Mucus Modulates Campylobacter jejuni Gene Expression in a Host-Specific Manner
Source: Front Microbiol. 2019 Jan 7;9:3215. doi: 10.3389/fmicb.2018.03215 (PMC6338021; doi:10.3389/fmicb.2018.03215)
Supplement: Supplementary file 5 [file Presentation_1.zip › Data Sheet 2.docx]

***Supplementary material***

**Antarctic krill are reservoirs for unique Southern Ocean microbial communities**

**Laurence J. Clarke*, Léonie Suter, Rob King, Andrew Bissett, Bruce E. Deagle**

*** Correspondence:** Laurence J. Clarke: [laurence.clarke@utas.edu.au](mailto:laurence.clarke@utas.edu.au)

**Figure S1.** Non-metric multidimensional scaling (nMDS) plot of bacterial communities from DNA extraction blanks, environmental and Antarctic krill (*Euphausia superba*) microhabitats using weighted (A) or unweighted UniFrac distance (B). Samples with DNA extraction yields less than 0.2 ng/μL are shown as open symbols. Extraction blank controls are most similar to digestive gland samples with low DNA extraction yields with weighted UniFrac distance, but more similar to higher yield samples with unweighted UniFrac distance, presumably due to cross-contamination. Faecal samples with low DNA yields show are widely dispersed over both ordinations, suggesting a low signal-to-noise ratio.

**A B**

**Figure S2.** Relative read abundance of bacterial classes (A) and orders (B) by sample type. Classes that were >1% reads or orders that were >2% reads in any one sample type are shown, the remaining taxa are pooled as ‘Other’.
