# Supplementary material for: Avian Intestinal Mucus Modulates Campylobacter jejuni Gene Expression in a Host-Specific Manner
Source: Front Microbiol. 2019 Jan 7;9:3215. doi: 10.3389/fmicb.2018.03215 (PMC6338021; doi:10.3389/fmicb.2018.03215)

Figure S1

MALDI-TOF mass spectrum of Sample-1 Oglycan PM (chicken)

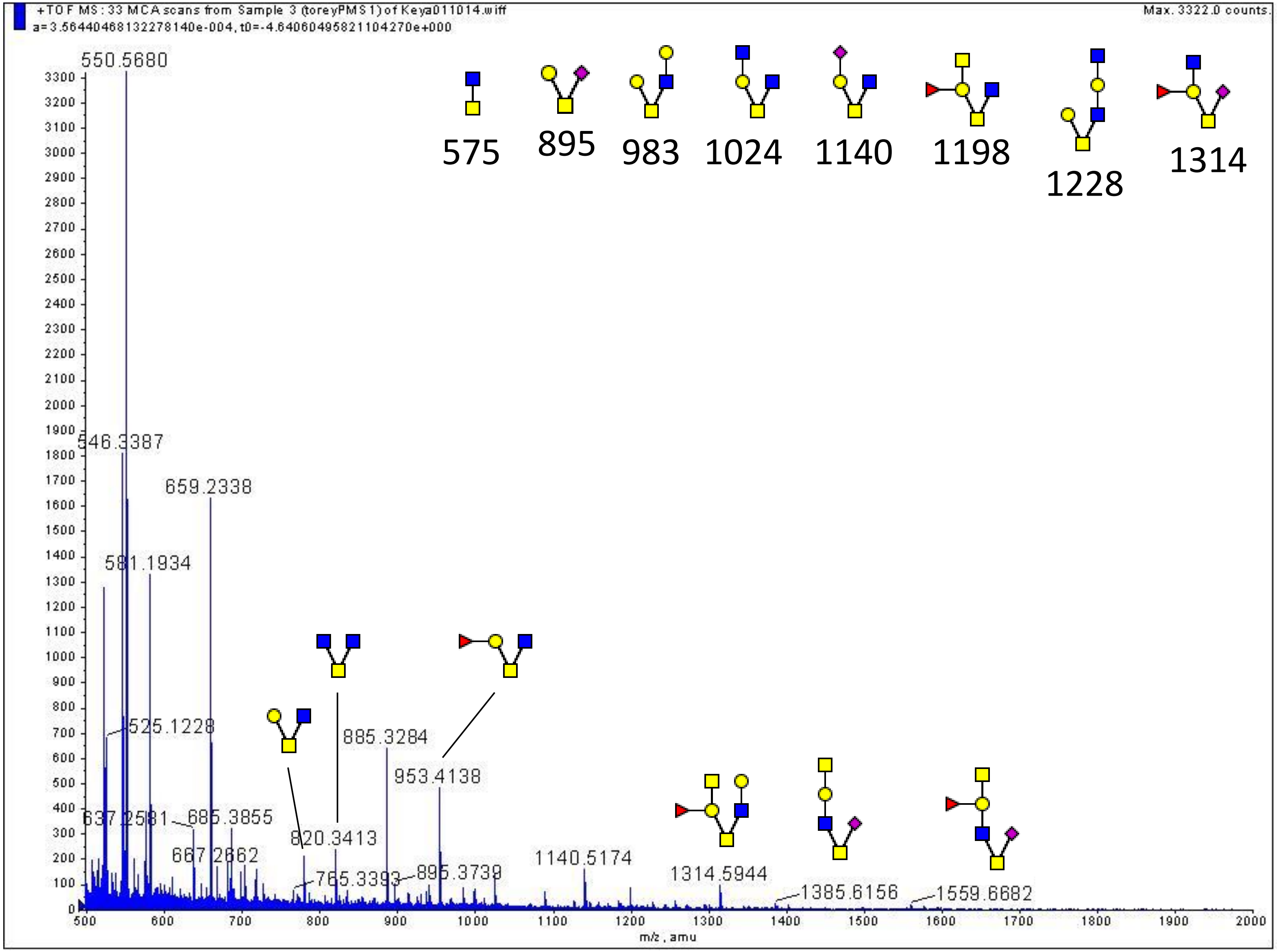

The structures are assigned that best fits the mass observed however there can be other isobaric species with different structure

MALDI-TOF mass spectrum of Sample-2 Oglycan PM (cow)

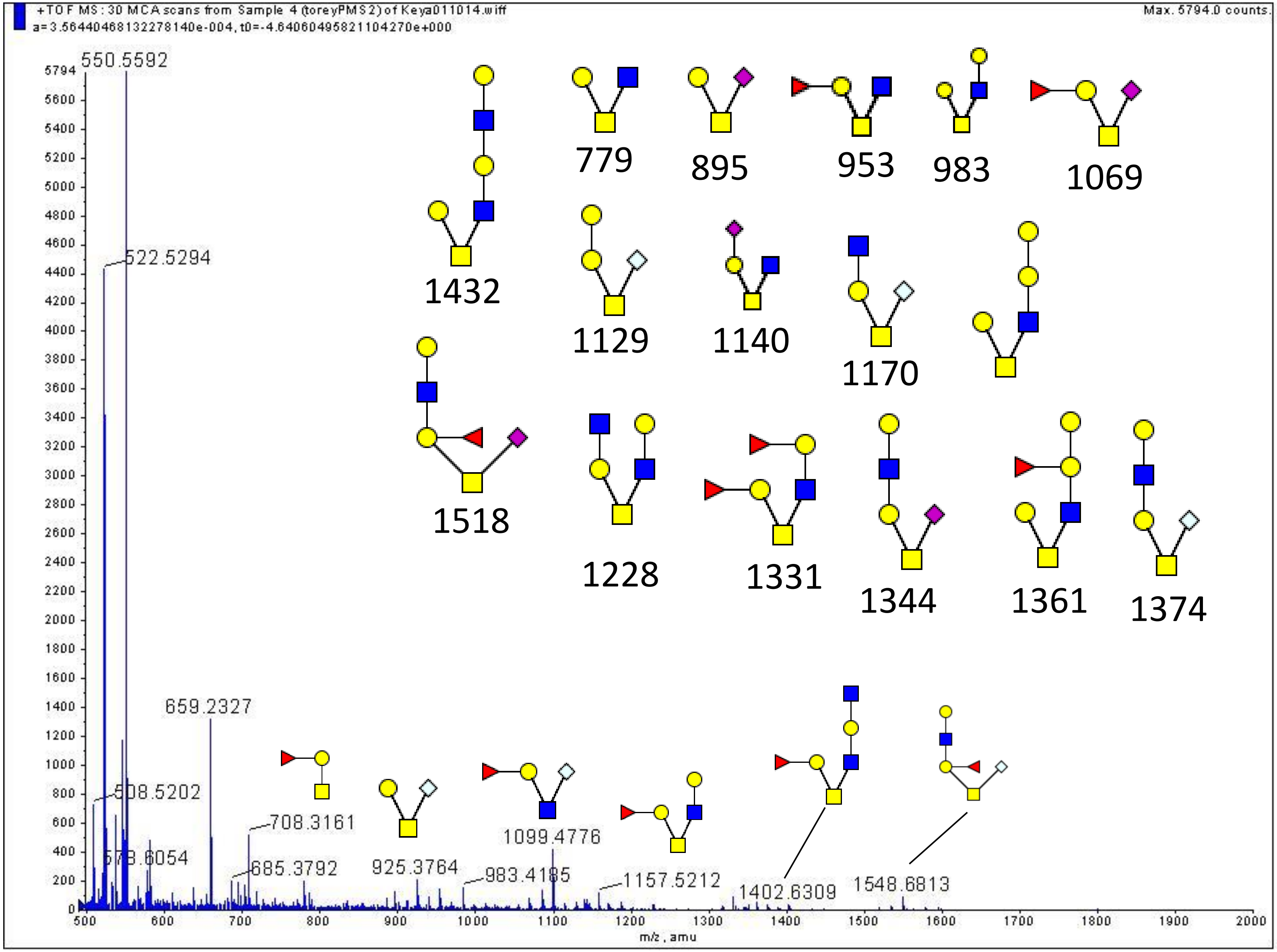

MALDI-TOF mass spectrum of Sample-3 Oglycan PM (pig)

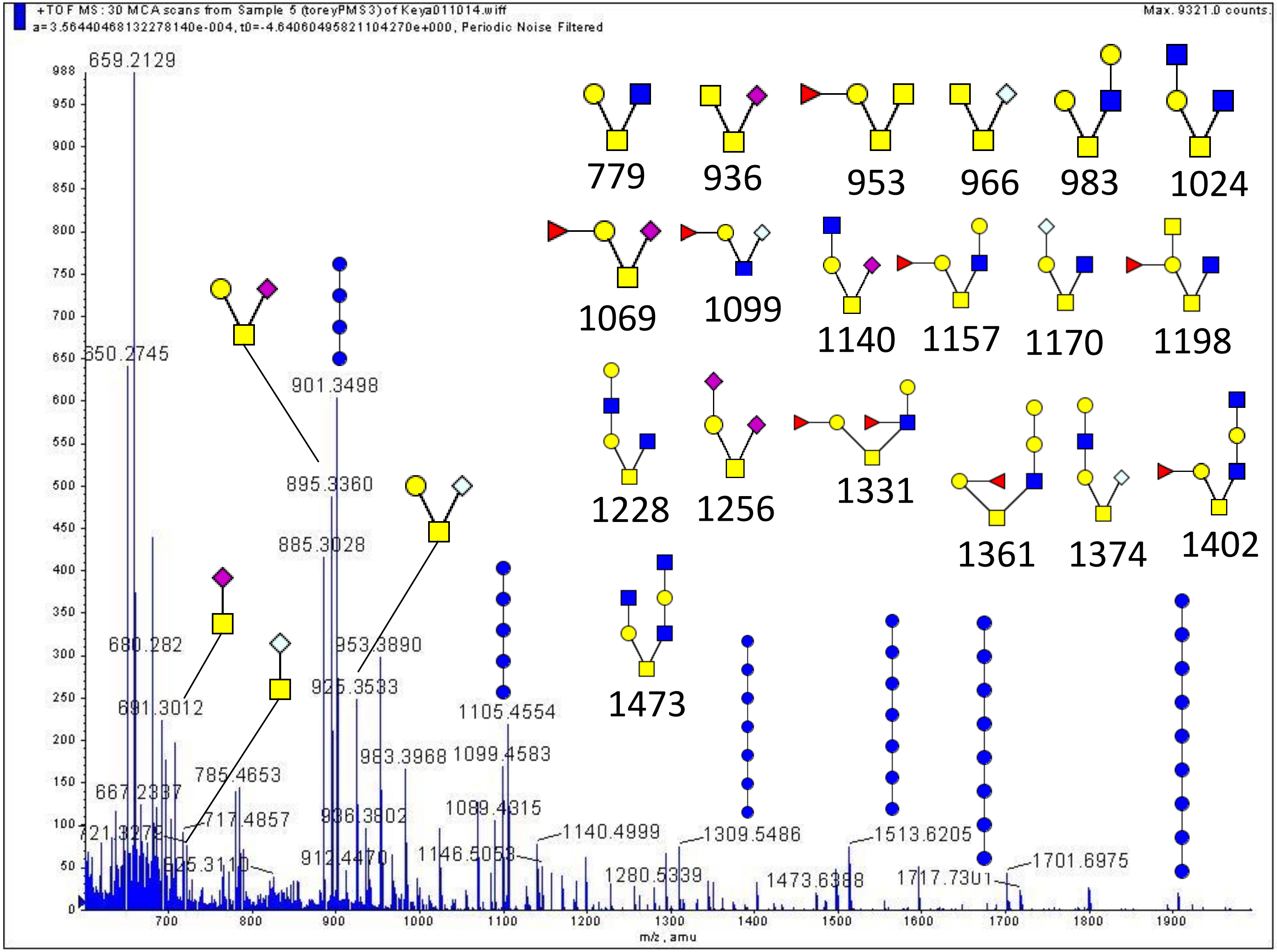

This sample also contain high amount of poly hexose (Glucose oligomers) Blue circles = Glc; Yellow =Gal, red triangle = Fuc, blue square = GlcNAc, yellow square = GalNAc, purple diamond = N-acetyl neuraminic acid, light blue diamond = N-glycolyl neuraminic acid

MALDI-TOF mass spectrum of Sample-4 Oglycan PM (sheep)

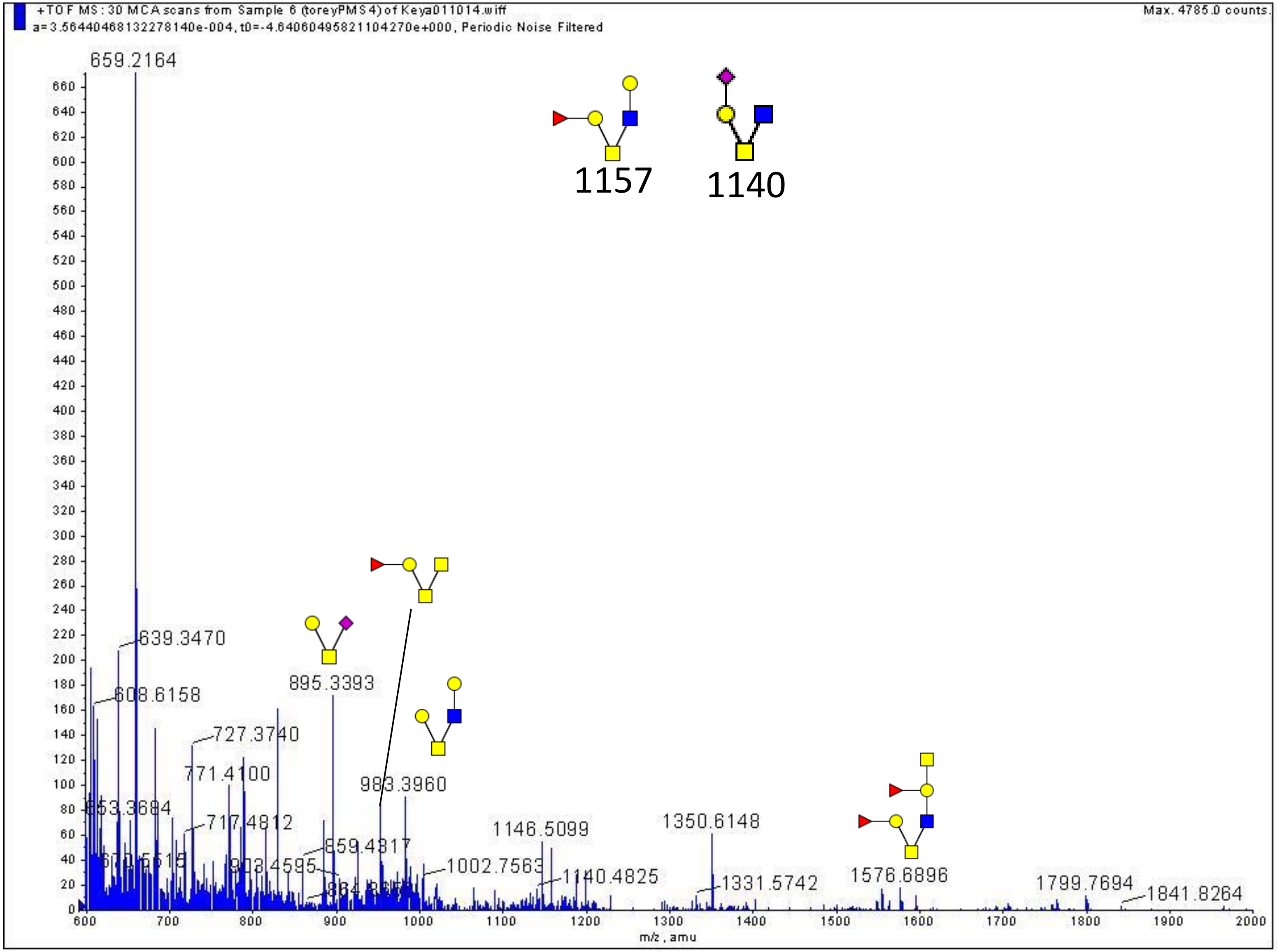

MALDI-TOF mass spectrum of Sample-5 Oglycan PM (Turkey)

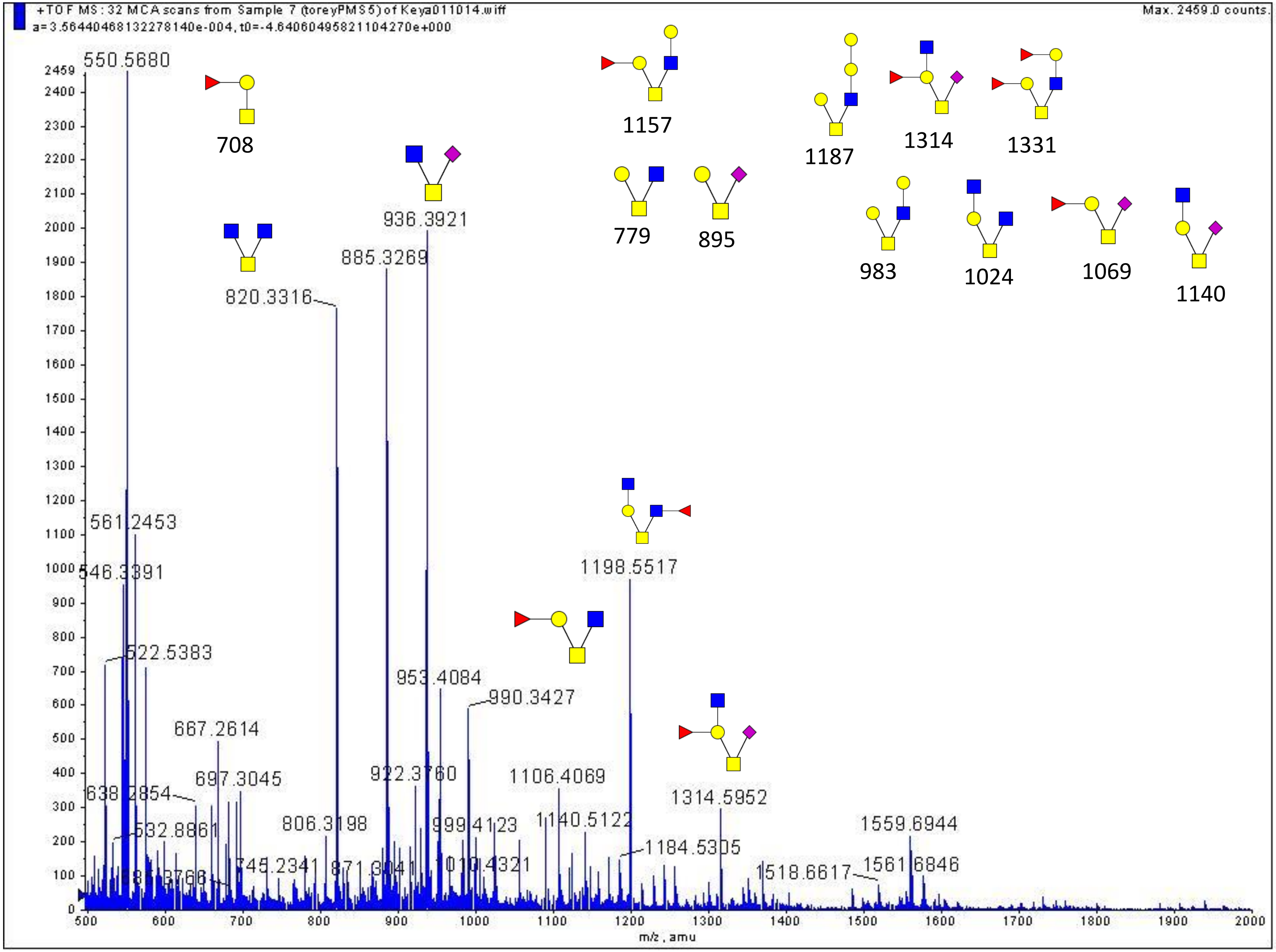

Supplement: Supplementary file 5 [file Presentation_1.zip › SF11.pdf]
