# Supplementary material for: Avian Intestinal Mucus Modulates Campylobacter jejuni Gene Expression in a Host-Specific Manner
Source: Front Microbiol. 2019 Jan 7;9:3215. doi: 10.3389/fmicb.2018.03215 (PMC6338021; doi:10.3389/fmicb.2018.03215)
Supplement: Supplementary file 5 [file Presentation_1.zip › SF33.pdf]

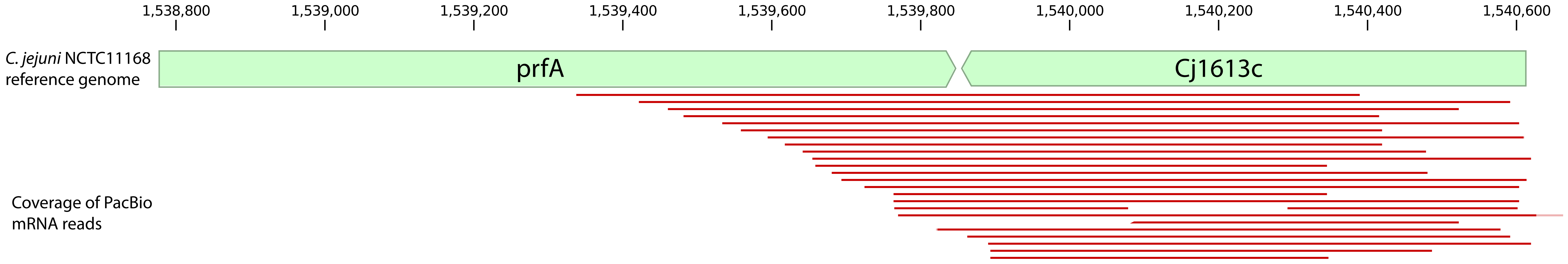

Figure S3. Sequence coverage map of C. jejuni ISO-seq mapped reads. Full length mRNA from C. jejuni 11168 grown, in chicken mucus was sequenced on the PacBio ISO-seq platform and mapped to the reference genome to identify full-length transcripts. Mapped reads show that full-length transcripts are part of transcriptional read-through events that span adjacent genes, leading to sense and antisense portions on single mRNA molecules. Genome location is indicated on top of figure, with gene names below (prfA and Cj1613c).
