# Supplementary figures and images for: Avian Intestinal Mucus Modulates Campylobacter jejuni Gene Expression in a Host-Specific Manner
Source: Front Microbiol. 2019 Jan 7;9:3215. doi: 10.3389/fmicb.2018.03215 (PMC6338021; doi:10.3389/fmicb.2018.03215)

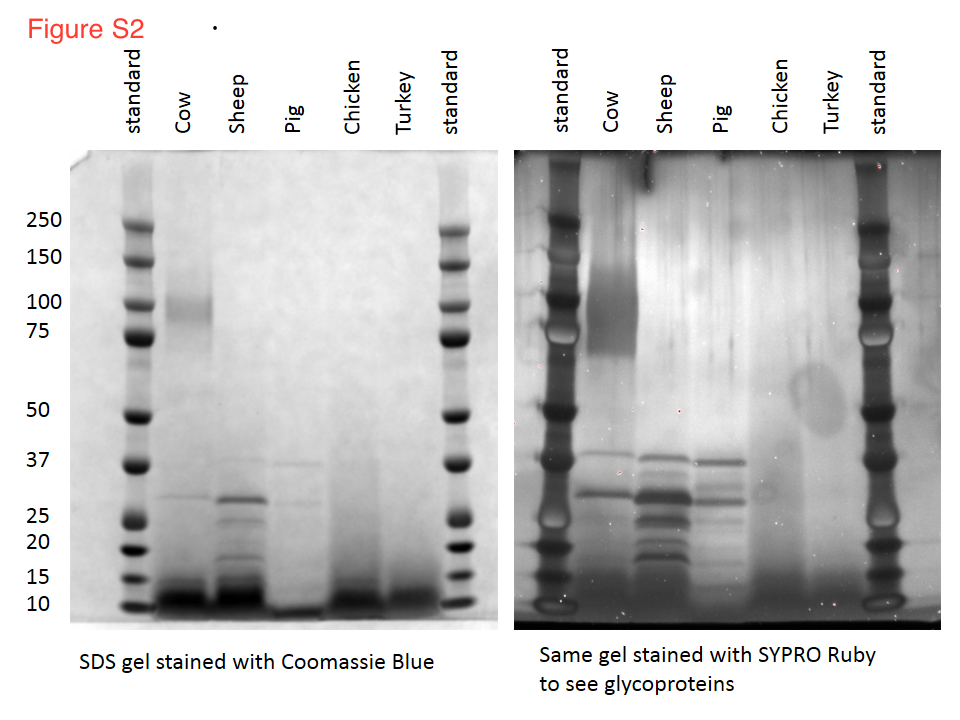

Supplement: Supplementary file 5 [file Presentation_1.zip › SF22.TIFF]
